# Human Stem Cell Use in Androgenetic Alopecia: A Systematic Review

**DOI:** 10.3390/cells12060951

**Published:** 2023-03-21

**Authors:** Katarzyna Krefft-Trzciniecka, Zuzanna Piętowska, Danuta Nowicka, Jacek C. Szepietowski

**Affiliations:** Department of Dermatology, Venereology and Allergology, Wrocław Medical University, 50-368 Wrocław, Poland

**Keywords:** stem cells, androgenic alopecia, female pattern hair loss, mesenchymal stem cell, follicle-derived stem cells, alopecia, male pattern hair loss, tissue regeneration, clinical application

## Abstract

Androgenetic alopecia is a condition that results in hair loss in both men and women. This can have a significant impact on a person’s psychological well-being, which can lead to a decreased quality of life. We conducted a systematic review to evaluate the efficacy of using stem cells in androgenic alopecia. The search was conducted in MEDLINE via PubMed, Web of Science, and Scopus databases. The review was performed on data pertaining to the efficacy of using different types of stem cells in androgenic alopecia: quantitative results of stem cell usage were compared to the control treatment or, different types of treatment for female and male androgenetic alopecia. Of the outcomes, the density of hair was analyzed. Fourteen articles were selected for this review. During and after treatment with stem cells, no major side effects were reported by patients with alopecia. The use of stem cells in androgenic alopecia seems to be a promising alternative to the standard treatment or it could play the role of complementary therapy to improve the effect of primary treatment. However, these results should be interpreted with caution until they can be reproduced in larger and more representative samples.

## 1. Introduction

The common cause of non-scarring alopecia among both men and women is androgenic alopecia (AGA). The disease manifests by progressive hair loss, usually in a pattern distribution. It can begin at any age after puberty, but the incidence increases with age. At the age of 70 and above, up to 42% of women and 80% of men present characteristic features of AGA [1]. The miniaturization of hair follicles and decreased hair density occur in the affected scalp area of patients with AGA. Terminal hair growth length is gradually reduced in this disease entity. Both women and men show an increase in short telogen hairs (<30 mm) over the course of the disease. This indicates that hair completes its life cycle in less than 6 months in PHL (pattern hair loss) patients [2]. Although the causes of miniaturization are unknown, genetic tendencies and androgen effects are thought to be associated with other factors that have not yet been clarified [3].

The hair of the human scalp plays an important symbolic role in determining one’s appearance and is an important sociocultural role. Hairstyles and lengths are distinctive features of an individual’s identity, and hair loss can lead to dissatisfaction with appearance, especially among women, and can have a negative impact on self-esteem. Hair loss is also associated with psychological and social difficulties in men [4,5].

At present, minoxidil and finasteride are the relevant only approved drugs by the US Food and Drug Administration (FDA). Dutasteride, despite not being an FDA-approved drug for androgenic alopecia, showed greater long-term effectiveness and safety versus finasteride in patients with male androgenic alopecia [6]. Low level laser light therapy (LLLLT) is the only approved device by the FDA to treat androgenetic alopecia [7]. Gupta et al. [8], in a systematic review with meta-analysis from 2022, came to the conclusion that the only treatment option with strong evidence, for both males and females with AGA is 5% minoxidil. AGA is a dynamic and progressive disease, therefore after initial diagnosis it is important to quickly apply not only a treatment that inhibits the progression of the disease but also one that can reverse the changes that have already occurred.

Stem cell-based therapies have recently gained considerable attention as potential novel treatments, focusing on the reactivation of hair follicle stem cells and thus improving the growth, regeneration, and development of hair follicles [9]. The sources of multi-potent stem cells with regenerative potential for hair follicles are adipose tissue, hair follicles from unaffected regions, blood, bone marrow, and Wharton’s jelly. There are two main types of stem cell transplants: autologous and allogeneic. The stem cells in autologous transplants come from the same person who will get the transplant, so the patient is their own donor. The stem cells in allogeneic transplants are from a person other than the patient, either a matched related or unrelated donor. Anudeep et al. proposed the classification of stem cells into adult stem cells and perinatal stem cells. Adult stem cells were divided into adipose tissue-derived stem cells (ADSCs), hair follicle-derived stem cells (HFSCs), and bone marrow-derived stem cells (BMSCs). In addition, ADSCs were divided into nanofat and stromal vascular fraction (SVF). HFSCs on autologous micrografts and cultured HFSCs. Perinatal stem cells included umbilical cord blood derivatives, MSCs from Wharton’s jelly, MSCs from amniotic fluid, and MSCs from the placenta [10]. Cell transplantation is one of the strategies to achieve the functional regeneration of hair follicles. Studies show that the injection of a mixture containing skin epithelial stem cells and mesenchymal stem cells can induce new hair follicles. Importantly, hair follicular stem cells (HFSCs) periodically switch between the active and inactive phases which enables keeping stem cell populations in the hair follicles stable; however, this ability is diminished with aging [11].

Hair follicles contain a variety of cell resources, such as melanocytic cells, epithelial cells, and stem cells from different developmental origins, which are capable of constantly renewing, distinguishing, and regulating hair growth and skin homeostasis [12,13]. The subjects of the greatest interest are two types of stem cells in the hair follicle. The first are HFSCs, which are in the region of the attachment of the arrector pili muscle and within the outer root sheath (ORS), in the region of the proximal end of the isthmus. Both regions are referred to as the “bulge.” The second type is dermal papilla cells (DPCs), which are responsible for controlling hair induction and growth as well as participating in the formation of new hair follicles [14]. Unlike scarring alopecia, in AGA, which belongs to non-scarring alopecia, there is no damage to HFSCs while progenitor cells are damaged [15]. That fact makes androgenetic alopecia potentially reversible. DPCs in the skin affected by androgenetic alopecia have reduced replicative potential and were also found to have changes in their shape, size, and loss of characteristic markers. The influence of dihydrotestosterone on DPCSs is considered a potential cause of senescence. Abnormally functioning DPCs produce inhibitory factors that suppress HFSCs. In turn, they lose the function of their stimulation [16].

Adipose tissue, in addition to its role in energy storage, is a well-known source of precursors and stem cells. The adipose tissue is a storage area for regenerative molecules. Indeed, a variety of regenerative products can be generated from adipose tissue, including nano fat, vascular stem cell fraction (SVF), MSCs, adipose-derived stem cell conditioning medium (ADSC-CM), and extracellular veins (EV) [10]. The stromal vascular fraction (SVF) refers to all the cellular elements of the adipose tissue and is formed from heterogeneous cell population components of this tissue: adipocytes, blood cells, endothelial cells, extracellular matrix, and the most essential elements which are adipose-derived stromal/stem cells (ASC) [17,18]. Multipotent stem cells derived from adipose tissues, stromal vascular cells derived from adipose (ADSVC), or regenerative cells derived from adipose (ADRC) refer to the newly used primary multipotent stem cells derived from the stromal vascular fraction. When these cells are cultured, they acquire additional characteristics and become a set of mesenchymal stem cells (MSCs) which are known as adipose-derived stem cells (ADSCs) [9,19,20].

Bone marrow is the main source of MSC. Many animal models showed the progression from the telogen phase to the anagen phase after the intradermal injection of mesenchymal cells from bone marrow (BM-MSC) and induced genes involved in hair regeneration [10,21]. Yoo et al. [22] studied the influence of MSCs from the umbilical cord and bone marrow on human hair proliferation in vitro. The researchers have attempted to cultivate hair follicle cells in vitro and implant them in the treatment area. The study showed that created DPLTs (dermal papilla-like tissues) have the same hair bulb structure inductive ability as natural DPSCs and that the transplantation of DPLTs can induce new hair follicles in athymic mice.

Studies using Wharton’s jelly stem cells on AGA patients have not been conducted. Their uniqueness and advantage over other mesenchymal cells are due to several of their features: non-invasive and non-painful derivation, large number of donors, no ethical concerns, high cell proliferation, and broad differentiation potential. In addition, they have negligible immunogens [23].

In recent years, there has been an intensive development of simple, outpatient stem cell isolation methods. At the same time, due to high availability and media publicity, they have become popular among both physicians and patients suffering from androgenetic alopecia. These are usually one-day procedures that do not require convalescence.

Along with the development of these techniques, a multitude of studies was published on the efficacy of stem cell-based treatments in AGA. However, no systematic review was performed. Therefore, this study aims to conduct a systematic review of studies assessing the efficacy of stem cell-based therapies in the treatment of AGA.

## 2. Materials and Methods

This systematic review was conducted to identify evidence on the efficacy of using stem cells in the treatment of AGA. To conduct this study, the MEDLINE, Web of Science, and Scopus databases were used. In addition, the reference list of included studies was also manually analyzed by the reviewers. This review was designed and conducted according to the PRISMA guidelines.

To retrieve articles, the following search query was used: (androgenic alopecia OR pattern hair loss) AND (stem cells OR ADSC OR ASC OR FDSC OR HFSC OR MSC). The search was conducted on 11 January 2023.

Inclusion criteria were randomized controlled trials (RCT), non-RCTs, case studies/series, and studies assessing treatments with stem cells in people with AGA. Pre-clinical models (animal studies), in vitro studies, narrative reviews, hypothetical cases, and observational studies were excluded. The exclusion criteria were as follows: full text not available, full text not available in English, studies with duplicate information published elsewhere, nonoriginal data such as reviews, commentaries, and editorials. In the case of studies from the same centers reporting possibly overlapping cohorts, the newest study was included. The inclusion criteria are reported as per the PICOS criteria and presented in Table 1.

Study identification and screening were made by two independent reviewers and discrepancies were resolved by consensus. One reviewer extracted data, while a second reviewer validated the extracted data. The following data were retrieved: study design, year of publication, percentage of female subjects, number of participants, age of participants, type of stem cells, evaluation method, and results.

## 3. Results

**Figure 1:**
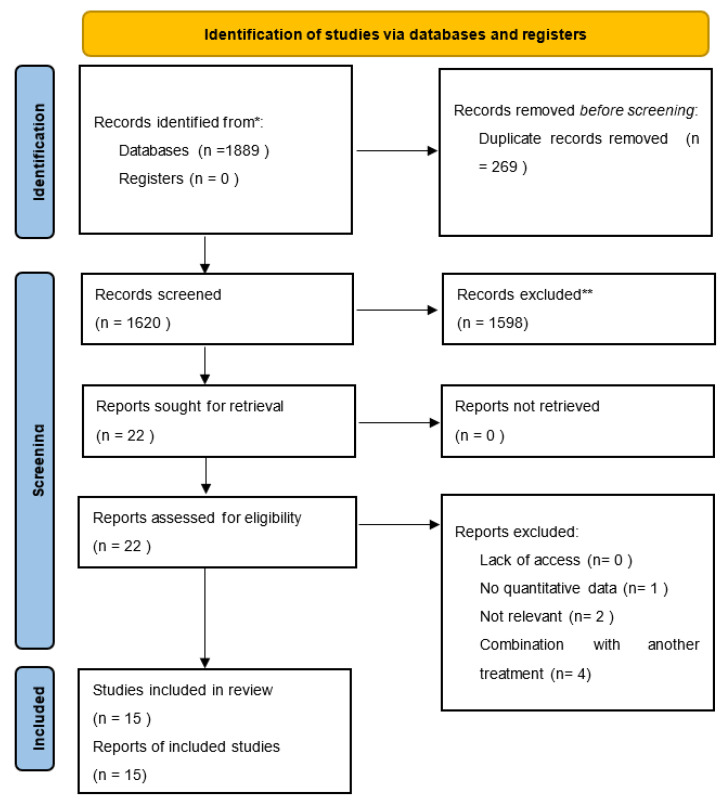
PRISMA flowchart of selected studies.

In the search, we identified 1889 records (685 in MEDLINE/PubMed; 289 in Web of Science; 915 in Scopus). Out of these, 210 duplicate records were removed. All titles and abstracts were reviewed, and 22 articles were assessed in full and reviewed for eligibility. Fifteen articles met the inclusion criteria and are summarized in this review. The selection process in presented in the PRISMA flowchart (Figure 1). The studies included in this review were conducted between 2015 and 2021. Studies were conducted in Italy (3), Egypt (2), Saudi Arabia (1), Japan (3), Spain (2), and South Korea (4). These studies included a total of 653 participants diagnosed with AGA. In total, 5 out of the 15 studies were reported as randomized controlled trials. Three were non-randomized controlled trials. Four were single-arm trials without a control group. Three were retrospective reports of a series of patients. The characteristics of included studies are shown in Table 2.

### 3.1. Studies Comparing the Use of Stem Cells with a Placebo

Of the 15 studies, 6 compared the use of stem cells with a placebo. Four of them compared HFSCs and two of them ADSCs.

Tak et al. [33] conducted a double-blind, randomized, placebo-controlled clinical trial. This study took place in the Republic of Korea and involved 38 patients (23.7% were females) diagnosed with AGA. Two groups were distinguished: the intervention group (IG; n = 19), in which participants received ADSC-CE (adipose-derived stem cell constituent extract) and the control group (CG; n = 19), in which participants received the vehicle placebo. Participants used ADSC-CE topical twice a day for 16 weeks. After 16 weeks, an evaluation based on phototrichogram showed that the total hair count was significantly lower in CG than in IG (13.95 ± 4.01 vs. 17.58 ± 4.13 counts per cm^2^ *p* = 0.009), although there was no significant difference in the hair thickness between the groups. During the course of the study, seven mild side effects were reported in five patients, which resolved without any medical intervention.

Another double-blind randomized controlled study was performed by Lee et al. [32] with 30 females and males aged 20–61 years, with MPHL (male pattern hair loss) or FPHL (female pattern hair loss). The treatment course consisted of the repeated application of ADSC-CM topically or normal saline (placebo) once per week for 12 consecutive weeks. At the endpoint, the hair density of the placebo group was 89.3 ± 3.79/cm^2^ and that of the ADSC-CM group was 102.1±4.09/cm^2^, showing a statistically significant difference (*p* < 0.05). No adverse events occurred during the duration of this study.

Gentile et al. [25] used human follicle stem cells (HFSCs) in 11 patients (38 to 61 years old) affected by AGA in stages 3–5, as determined by the Norwood-Hamilton classification scale. He used the system Rigenera^®^ which obtains stem cells from patients’ scalp biopsies without culturing. In patients with hair loss in the frontal and parietal areas, the HFSC injections were administered exclusively to the front scalp, and placebo injections (i.e., normal saline) were administered to the parietal areas. Similarly, in patients with hair loss limited to the parietal and vertex regions, HFSCs were injected into the parietal region and placebos were injected into the scalp vertex. The hair density after 23 weeks increased by 29% ± 5% for a treated area and by less than 1% for the placebo area. They hypothesized that stem cells can improve the formation of new follicles. No major side effects were reported.

Another study conducted by Gentile et al. [27] in 2019 with 21 participants demonstrated that the average hair density among patients from the treatment group with HFSCs increased by more than 30 ± 5.0% and from the placebo group it increased by less than 1% 12 weeks after the treatment. There was no mention of the occurrence of any side effects.

Furthermore, in 2020 Gentile et al. [31] used autologous micrograft injections with HFSCs 3 times 45 days apart on a group of 27 participants and reported an improvement in the mean hair count after 58 weeks (58 weeks vs. 0 weeks). The mean increase (vs. baseline) was 18.0 hairs in the treated area while the control area displayed a mean decrease of 1.1 hairs. There was no mention of the occurrence of any side effects.

Tsuboi et al. [24] studied the effectiveness of autologous cell therapy with dermal sheath cup cells (DSCs) to treat MPHL and FPHL. Subjects received injections with three concentrations of DSC cell suspensions (7.5 × 106, 1.5 × 106 and 3.0 × 105,134 cells) and a placebo (each in a volume of 1 mL) into 4 randomly allocated injection sites. The effectiveness was assessed by imaging of the phototrichogram before injection and 3, 6, 9, and 12 months later, and the hair density and hair diameter were measured using an image analysis system. The total hair density and cumulative hair diameter increased significantly for 6 months and 9 months at the DSC cell injection site with a low dose compared to placebo. In 14 cases, mild adverse reactions such as erythema, purpura, and small hemorrhages were observed at the injection sites.

### 3.2. Studies Analyzing the Efficacy of Stem Cell Treatment Alone

Of the 15 studies, 4 analyzed the use of stem cells treatment alone. Two of them assessed HFSCs and two of them ADSCs.

The study carried out by Zari et al. [34] in a group of 140 consecutive adults with confirmed AGA sought to examine the efficacy of autologous cellular micrografts which contain HFSCs. Efficacy was evaluated 1–6 months after treatment by analyzing the change of trichometry parameters, which showed that depending on the scalp region there was an increase in the mean hair density by 4.5–7.12 hair/cm^2^. No side effects were reported.

Ruiz et al. [35] performed a study in a group of 100 participants with HFSCs from autologous micrografts obtained through Rigenera^®^ micrografting technology. The outcomes were confirmed by the TrichoScan^®^ and showed that the mean increase in the total hair density was already 30% ± 3.0% after 2 months of treatment compared with baseline values for the treated area. In addition, scalp dermoscopic analysis also showed an improvement in hair density after both 4 and 6 months following treatment. No side effects have been reported.

Kim et al. [29] reported nine patients who were suffering from AGA with single transplantation of autologous SVF in the upper scalp. Hair density of the ADSCs-treated side was significantly increased after 3 and 6 months of transplantation compared to the non-treated side (*p*  =  0.01 and *p*  =  0.009 per each). There was no mention of the occurrence of any side effects.

El-Khwalawany et al. [26] analyzed the efficacy of the non-enzymatic vascular fraction (SVF) in 30 patients with AGA. Patients received one SVF injection and a single 6-month follow-up session. The number of hairs increased from 130.87  ±  14/cm^2^ to 151.93  ±  22.36/cm^2^. Patients were asked about experienced pain during and after the procedure and 21 reported mild pain and 9 reported moderate pain. No one reported severe pain. Other than that, no other side effects were reported.

David Perez Meza et al. [19], in their retrospective observational case-series study, analyzed the results of using SVF-enhanced adipose tissue among 6 men and women aged 18–55 years with MPHL and FPHL. In this pilot case series, a mean increase of 31 hairs/cm^2^ of the scalp (represents a 23% relative percentage increase) was documented in patients undergoing treatment of fat plus. One side effect was described, and it was a hematoma in the hairline area after fat injection. The patient did not require medical intervention.

A retrospective, observational study of outcomes in 27 patients with FPHL treated with ADSC-CM was also performed by Shin et al. [28] The application of ADSC-CM with a micro-needle roller once per week showed efficacy in treating FPHL after 12 weeks of therapy. The hair density increased from 105.4 to 122.7 hairs/cm^2^ (*p* < 0.001). The hair thickness increased from 57.5 lm to 64.0 lm (*p* < 0.001). No serious side effects were observed in any patient.

### 3.3. Studies Comparing Stem Cells and/or Other Treatments

Of the 15 studies, 3 compared the use of stem cells to another treatment.

Elmaadavi et al. [36] studied the safety and efficacy of autologous bone marrow-derived mononuclear cells (BMMCs) including stem cells and follicular stem cells (FSCs) in 40 patients with alopecia areata (AA) and AGA. From 20 patients with AGA, 10 received BMMCs and 10 received HFSCs injections intradermally. The evaluation by immunostaining and digital dermatoscopy showed an increase in hair thickness as well as hair density after six months of single injection therapy in all groups of patients with no significant difference between both methods in either type of alopecia (*p* = 0.426). The average improvement percentage in AGA patients was 52 ± 28 in subjects receiving autologous BMMC and 42 ± 27 in subjects receiving autologous FSC, with no statistically significant difference. Elmaadavi et al. reported fatigue and chills in several patients after the administration of a granulocyte colony-stimulating factor. Moreover, 80 percent of the subjects developed bone pain and hematomas.

Narita et al. [37] evaluated the effectiveness of ADSC-CM in 21 male and 19 female patients. The ADSC-CM treatment was evaluated in subpopulations depending on whether or not finasteride was administered. Patients received ADSC-CM intradermal injections each month for 6 months, followed by follow-up assessments before and after 2 and 6 months. In this study, the density of hair in all the groups increased considerably. The density of hair increased from T0 (time of the intervention) to T (status after 6 months after treatment) (*p* < 0.001). However, specific results were reported only for 2 patients. There was no mention of the occurrence of any side effects.

In a study published in 2015, Fukuoka and Suga [30] compared the effectiveness of ADSC-CM in male patients with AGA who took finasteride and those who did not, and among female patients without finasteride. Patients received 6 sessions of ADSC-CM injections every 3–5 weeks. The number of hairs based on a trichogram increased significantly after treatment in both male (n = 11) and female (n = 11) patients. The mean increase in the number of hairs was 29 ± 4.1 in male patients and 15.6 ± 4.2 in female patients. No significant difference was observed between men and women. In male patients, groups with (n = 6) and without (n = 5) finasteride administration were compared. Fukouka and Suga reported that the most common complication during the procedure was pain during and after injections with ADSC-CM.

## 4. Discussion

This is the first systematic review on the use of different types of stem cells in the treatment of female and male AGA. In total, 15 studies, in which 653 patients were involved, provided a comprehensive view of the effects of autologous stem cells in the treatment of AGA. This review shows the impact of stem cells from different origins on the density of hair. The main conclusion of the analyses of the studies we selected is that stem cell treatments have positive effects on the density of hair regardless of its origin.

We analyzed databases of privately and publicly funded clinical trials conducted around the world (clinicaltrials.gov, accessed on 15 March 2023) and are awaiting the results of many of them.

Stem cells used in the treatment of androgenetic alopecia are mainly derived from two sources: hair follicles and adipose tissue. Hence, almost all studies relate specifically to these cells. Only Elmaadavi et al. [36] studied the efficacy of BMSCs in a group of 10 people with AGA, while the available research on stem cells from Wharton’s jelly refers only to alopecia areata. We made the decision to compare hair density even though the more defining feature of AGA is hair thickness because only a few studies measured this parameter, while everyone studied hair density. In androgenetic alopecia, the diameter variation of more than 20% of hair in the androgen-dependent region is considered to be a major diagnostic standard of androgenetic alopecia [38]. Of note, 2 studies included over 100 patients and reported increased mean hair density. Zari et al. involved 140 participants in their study and in 66, 4% reported a significant improvement in hair density in the frontal region of the head.

In the papers we reviewed, the main side effects were reported by Elmaadavi et al., who declared that some patients after granulocyte-colony stimulating factor reported fatigue and chills. Eighty percent of subjects also suffered from bone pain and hematoma. All symptoms were managed with painkillers and anti-inflammatory drugs. In the same study, for 20% of patients treated with FSC, the only side effect was scalp irritation, which was treated with emollients. For the reasons mentioned above, the effectiveness of different stem cells needs to be compared, as treatment with them is associated with different recovery. Treatment with BMSCs is more absorbent for doctors and patients.

It seems interesting that several researchers have confirmed differences between the effectiveness of stem cells according to gender. Elmaadavi et al. reported a larger improvement in females compared to males after both BMMC and FSC therapies (*p* = 0.016 and 0.008, respectively), but no significant difference was found between both types of therapy. Zari et al. showed, in a trichoscopy examination, that the most notable effect was increased hair density in men, whereas in women it was improved hair thickness and reduced yellow dots. On the other hand, Tsuboi et al. [24] reported that men and women achieved similar results in their study. They concluded that since cell therapy has similar results in both sexes, it will be an excellent treatment alternative for women, who have more limited treatment options compared to men.

An interesting result presented by Tsuboi et al. [24] is that treatment with HFSCs was more effective in patients older than 51 years and in those with moderate disease severity (Hamilton grade III, IV and Shiseido grade 3, 4). It was suggested that this may be due to the fact that older patients may have a higher number of inactive resting hair follicles (telogen hair), so the injection of DSC cells showed a more notable improvement in the induction of hair growth.

The involvement of inflammatory cells (neutrophils and macrophages) in inducing hair follicle anagen and the relationship between hair follicle stem cell activation and inflammation remain further topics of discussion. [39] It is possible that the stem cell products being tested cause subtle inflammation that could contribute to transient hair growth.

We identified several limitations in the current research. Owing to the heterogeneity of the studies in terms of the demographic characteristics, treatment methods, and final evaluations of the subject, performing a meta-analysis was not feasible. The results we present should be interpreted with an awareness of their limitations.

Important limitations of the evidence are that the studies present different treatment protocols, with different types of stem cells and different amounts of sessions, duration of follow-up, etc. The researchers used different devices to evaluate the effects at different intervals. The criteria for the exclusion and inclusion of patients in the studies were also varied. It is also worth noting that patients at various stages of the disease, including those at very advanced stages, were enrolled in the studies. Moreover, it is widely known that many factors affect the quality of the stem cells administered, and not all studies reported information on how they were collected and prepared. Notably, only 9 of the 15 papers presented had a control group, and only 3 of those 9 studies compared the use of stem cells with another treatment. Six studies compared stem cells with a placebo. All controls should include products from other cellular sources, such as primary fibroblasts/keratinocytes, to confirm the necessity of stem cell use and exclude the contribution of buffers, media, and other components to treatment efficacy. This is an important limitation of those papers. Since many commonly used treatments for hair loss have no proven statistically significant effect, it is important to compare new therapies with other available effective treatments. In the case of the scientific papers described above, four of them evaluate the effect of stem cells only, which calls into question the usefulness of these studies.

## 5. Conclusions

According to the results of this review, the use of stem cell injections in female and male AGA appears to be a promising treatment option. Future research should focus on improving clinical research design, conducting more studies with stem cells in women, and establishing best practices in injection sites, cell types, sessions, and frequency. It is important to create a standardized method for collecting, preparing, and injecting stem cells. While there are numerous reports on ADSCs and HFSCs for the treatment of AGA, further studies with BMSCs and the initiation of studies with stem cells from Wharton’s Jelly are necessary to begin considering them as a therapeutic option. It would be helpful to conduct a study comparing the effectiveness of different types of stem cells for the treatment of AGA, as the ease of obtaining them varies widely. We highlight the need for studies investigating this because currently available therapies for AGA are unsatisfactory, and there is a demand for new treatment strategies.

## Figures and Tables

**Table 1:** PICO criteria.

**P**atients = women and men with androgenic alopecia	Descriptions: female pattern hair loss, male pattern hair loss, female androgenic alopecia, male androgenic alopecia, alopecia in women, alopecia in men.
**I**nterventions = treatment with stem cells	Descriptions: ADSC, HFSC, BMSC.
**C**omparison	Descriptions: placebo, different treatments.
**O**utcomes	Objective measurement of hair density.

ADSC, adipose-derived stem cell; BMSC, bone marrow stromal cell; HFSC, hair follicular stem cells; PICO, patient/population, intervention, comparison, and outcomes.

**Table 2:** Characteristics of included studies.

Author, Year	Design Country	N, Age (Year) F (%)	Duration and Type of Treatment	Progression of the Disease (Scale)	Assessment	Hair Density
Tsuboi et al. [24], 2020	RCT Japan	65 33–64 23	HFSCs 1 session n/r injections	N-H 3-6 S 3-6	P, hair density, hair diameter, trichogram	IMPROVEMENT: total hair density increased vs. placebo (*p* < 0.025), an increase was greater in people ≥51 years of age
Gentile et al. [24], 2017	NRCT Italy	11 38–61 0	HFSCs 1 session subcutaneous injections	N-H 3-5	P	IMPROVEMENT: hair density increased by 29% ± 5% for a treated area and by less than 1% for the placebo area
Elmadaavi et al. [25], 2018	RCT Egypt	20 10–50 (26 ± 8) 60	BMSCs and HFSCs 1 session intradermal injections	n/r	P, dermosopic examination, digital dermoscopic, and histological examination	IMPROVEMENT: increase in hair density of 52 ± 28 in subjects receiving BMMC and 42 ± 27 in subjects receiving FSC with a non-statistically significant difference
Zari et al. [26], 2021	single arm, non-controlled Saudi Arabia	140 18–65 (mean age 32.1) 80,7	HFSCs 1 session subcutaneous injections	S 2–4, N-H 2–4	P, trichoscopy (TrichoScan^®^)	IMPROVEMENT: hair density increases by +4.5 to 7.12 hair/cm
Narita et al. [27], 2020	NRCT Japan	40 23–74 47,5	ADSCs-CM 6 sessions intradermal injections	L 1-2, N-H 2–6	P, trichoscopy (TrichoScan^®^)	IMPROVEMENT: increased hair density from T0 to T6 (*p* < 0.001)
Ruiz et al. [28], 2019	single arm, non-controlled Spain	100 n/r n/r	HFSCs 1 session n/r injections	n/r	P, trichoscopy (TrichoScan^®^)	IMPROVEMENT: increased hair density of 30% ± 3.0%
Gentile et al. [29], 2019	ROCA Italy	21 25–72 28,2	HFSCs 2 sessions subcutaneous injections	L 1-2, N-H 2-5	P	IMPROVEMENT: increased hair density of 30 ± 5.0% for the treatment group and less than a 1% increase for the placebo
Kim et al. [29], 2021	single arm, non-controlled Republic of Korea	9 43–64 (53 ± 1.22) men: 51.5 ± 3.43; women 55,5	ADSCs 1 session subcutaneous injections	L 1-3, N–H 4-5	P	IMPROVEMENT: hair density increased compared to the non-treated side (*p* = 0.01 and *p* = 0.009 per each); density increased in the treated site by 48.11% as compared to the non-treated site density of 35.48%
Fukuoka et al. [30], 2015	NRCT Japan	32 20–70 40,2	ADSCs-CM 6 sessions intradermal injections	n/r	P, trichoscopy (TrichoScope)	IMPROVEMENT: the mean increase in hair density was 29 ± 4.1 in male patients and 15.6 ± 4.2 in female patients
El-Khalawany et al. [26], 2022	single arm, non-controlled Egypt	30 21–45 (30.1 ± 6.3) 53,3	ADSCs 1 session intradermal injections	L 1-3, N-H 1–6	P, trichoscopy	IMPROVEMENT: hair count/cm^2^ showed a high statistically significant increase from 130.87 ± 14/cm^2^ before the study to 151.93 ± 22.36/cm^2^ at the 6-month follow-up visit with a 16.09% improvement
Shin et al. [28], 2015	ROCS Republic of Korea	27 22–69 (41.9 ± 13.4) 100	ADSCs-CM 12 sessions topical wit micro-niddle roller	L 1	medical records and phototrichographic images were analyzed	IMPROVEMENT: hair density increased from 105.4 to 122.7 hairs/cm^2^ over the 12 weeks of treatment (*p* < 0.001), representing an increase of 16.4%.
Gentile et al. [31], 2020	RCT Italy	27 n/r 37	HFSCs 3 sessions subcutaneous injections	L 1-2, N-H 2-5	P, phototrichograms	IMPROVEMENT: an increase of hair count and hair density, respectively, of 18.0 hairs per 0.65 cm^2^ and 23.3 hairs per cm^2^ compared with the baseline, while the control area displayed a mean decrease of 1.1 hairs per 0.65 cm^2^ and 0.7 hairs per cm^2^ (control vs. treatment: *p* < 0.0001)
Perez Meza et al. [19], 2017	ROCS Spain	6 18–55 11,1	ADSCs 1 session subcutaneous injections	L 1-3, N–H 2-6	P, trichoscopy (FotooFinder^®^)	IMPROVEMENT: the mean increase of hair density was 31 hairs/cm^2^ of (represents a 23% relative percentage increase)
Lee et al. [32], 2020	RCT Republic of Korea	30 20–61 (46.6) 50	ADSCs-CM topical 12 weeks, 1 session weekly	n/r	P, phototrichogram	IMPROVEMENT: the hair density of the placebo group was 89.3 ± 3.79/cm^2^ and that of the ADSC-CM group was 102.1 ± 4.09/cm^2^, showing a significant difference (*p* < 0.05)
Tak et al. [33], 2020	RCT Republic of Korea	38 45.3 23,7	ADSCs-CM topical 16 weeks twice daily	n/r	P, phototrichogram	IMPROVEMENT: in the treatment group the mean hair density and thickness increased by 28.1% and 14.2% by 16 weeks, which were 3.95 and 2.25 times those in control group using the vehicle placebo

F, female; L, Ludwig scale; N-H, Norwood-Hamilton scale; S, Shiseido scale; N, number of participants; NRCT, non-randomized clinical trial; P, photography with baseline evaluation; RCT, randomized controlled trial; ROCS, retrospective observational case series study; S, Sinclair scale; n/r, non-reported.

## Data Availability

All data used is included in this review.
